# Executive Stress Management: Physiological Load of Stress and Recovery in Executives on Workdays

**DOI:** 10.3390/ijerph15122847

**Published:** 2018-12-13

**Authors:** Beatriz Crespo-Ruiz, Shai Rivas-Galan, Cristina Fernandez-Vega, Carmen Crespo-Ruiz, Luis Maicas-Perez

**Affiliations:** 1 Physical Activity and Sport Education Department, Faculty of Sport Sciences of Toledo at University of Castilla-La Mancha, 45002 Toledo, Spain; info@luismaicas.com; 2 Freedom & Flow SL, 28050 Madrid, Spain; shai.rivas@freedomandflowcompany.com (S.R.-G.); Cristina.fernandez@freedomandflowcompany.com (C.F.-V.); carmen.crespo@freedomandflowcompany.com (C.C.-R.)

**Keywords:** stress, stress management, human resources, executives, physiology, health, technology, business

## Abstract

*Objective*: The use of high-performance sports technology to describe the physiological load of stress and the quality of recovery in a population of executives during the workday. *Methodology*: Heart rate variability values were recorded during 48 h from which the relationship between stress/recovery quality (stress balance) was obtained for three differentiated time slots: work, after work, and night in a workday. *Results*: We observed a negative stress balance during the 24 h of measurement in the course of a workday, being negative at work and after work, and positive at night. The stress generated or maintained outside working hours correlates significantly with a lower quality of recovery during the 24 h workday. *Conclusions*: It is necessary to prioritize strategies that help improve stress management in executives through the improvement of tools and strategies that mainly promote greater relaxation outside working hours.

## 1. Introduction

Stress is defined as the physical and mental responses of the body and adaptations to real and perceived changes and challenges in life. Stress is, for many, considered as the silent disease of the 21st century. However, it was not officially classified as a disorder or alteration to take into account differently to anxiety until the 5th edition of the Diagnostic and Statistical Manual of Mental Disorders (DSM) by the American Psychiatric Association published on 1 October 2016.

Nowadays society faces the great challenge of adapting to a continuous rate of change, connected to a large number of internal and external stimuli that, poorly managed, can become important daily stress triggers, with the physical and mental consequences that all of this involves in and out of working time. To this effect, there are several challenges that companies and governments must face when it comes to promoting health and well-being. Actually the increase of pathologies, such as stress, anxiety, and/or depression, have led to financial losses up to €136 million in companies reaching percentages of more than 75% of workers in the European Union who suffer daily stress being already more than 20% the working age population that suffer serious health problems, such as the chronic stress syndrome known as burnout [1].

In this sense, and due to the multifactorial nature of stress, other factors such as poor rest, physical inactivity, overweight, and obesity are increasingly present in today’s companies, aggravating and increasing the risk of suffering cardiovascular diseases, immunological, as well as chronic diseases susceptible to reduce the quality of life of the individual [2,3,4], which affects not only at an individual level, but also at social and professional levels [5]. In this regard, and according to data from the European Heart Network, Spain is in the top ten European countries with a more sedentary lifestyle among adults; 42% of those over 18 say they do not do any physical activity during the week, against 6% in Sweden or 7% in Finland. In addition, data from the European Foundation for improving living and working conditions confirm that Spaniards spend an average of 1720 h a year at work [6].

Among the factors that currently have the greatest potential to become stressors, understood as stress triggering situations, could be any direct or indirect, external or internal stimulus (physical, chemical, acoustic or somatic, or sociocultural), that favors a disruption in the dynamic equilibrium of the organism (homeostasis) we can find environmental factors (work, family, work meetings, corporate culture, climate, environment, etc.) as those related to lifestyle (level of practice of physical activity, eating habits, and emotional management, among others) [7].

On a psychological level, a recent study detected through univariate and multivariate statistical analysis of the risk prediction score of cardiovascular disease (CVD), and of the general welfare of WHO, that a combination of general risk factors and organizational factors contribute to increase the risk and well-being of CVD, with a direct and inversely proportional relationship between work stress and the welfare index [8]. In addition, a study carried out with a sample of 2991 German and Chinese students where a lifestyle for positive mental health (PMH) and mental health problems (MHP) was analyzed through the Positive Mental Health Scale and a version of 21 items on the scale of anxiety, depression, and stress, obtained as results the importance of following a healthy lifestyle to improve psychological well-being and develop fewer mental health problems. Factors such as BMI, frequency of physical and mental activities, frequency of alcohol consumption, smoking, vegetarian diet and the irregularity of social rhythm were considered in this study to consider the lifestyle of the subjects [9].

**Figure 1:**
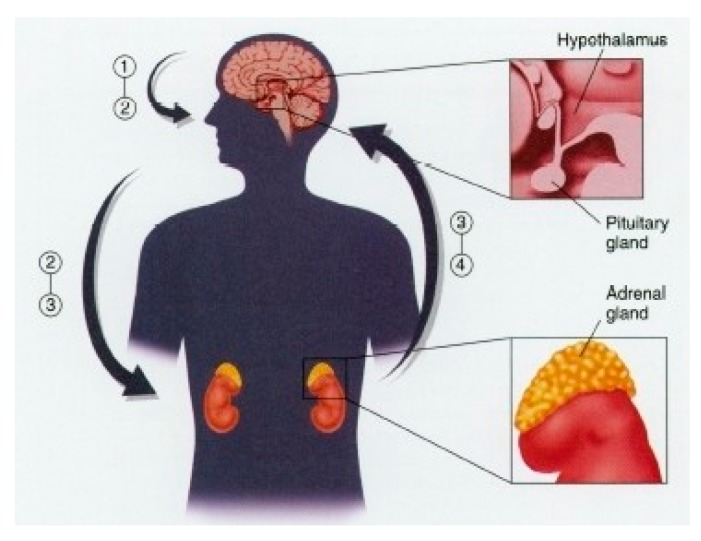
Neuroscience applied to stress control.

Physiologically, the exposure to one or several stressors’ stimuli triggers the response of the sympathetic nervous system (SNS), activating the hypothalamic pituitary adrenal axis in charge of releasing the hormone corticotropin (ACTH) that acts directly on the pituitary gland to secrete adrenocorticotropin, thereby increasing the levels of cortisol, cortisone, epinephrine, and norepinephrine into the blood. This chain reaction provoked by a stressor stimulus is responsible for increasing the levels of brain and blood glucose, heart rate (HR), and blood pressure, among others (Figure 1) [9].

It is important to comprehend the physiological process that triggers stress to better understand the type of strategies that will be necessary to be implemented in stress management. Additionally, the response of our body to various stressors corresponds to a “mechanism of escape” whose magnitude of enrollment; both neuroendocrine and physiological depend on the duration and specific needs of the body. Its implementation not only involves a high energy expenditure, but also the release of hormones and substances that generate an over-excitation of the nervous system, which, maintained over time, can lead to an intensification in cellular aging, oxidative stress, inflammation of tissues, and cardiometabolic problems, among others [10].

To know the levels of stress in the body from a physiological point of view there are two non-invasive methodologies: the measurement of cortisol levels through saliva [11] and the recording of heart rate variability (HRV) understood as the variation between two consecutive beats: the greater the variation, the greater the parasympathetic activity [12] through devices recording the HR.

**Figure 2:**
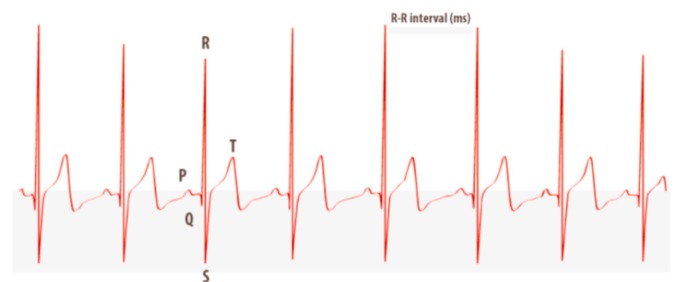
HRV means the variation in time between consecutive heartbeats.

Studying the heart by HRV (Figure 2) provides us with a vast amount of information about our body. From beat to beat, heart rate is constantly changing to meet the needs of life. HRV means the variation in time between consecutive heartbeats. It is universally accepted as a non-invasive marker of autonomic nervous system (ANS) activity. A variety of physiological phenomena affect HRV, including:

- Inhalation and exhalation, control of breathing
- ANS adjustments
- Hormonal reactions
- Metabolic processes and energy expenditure
- Physical activity, exercise, and recovery from physical activity
- Movements and changes in posture
- Cognitive processes and mental load
- Stress reactions, relaxation, and emotional reactions

Heart rate variability increases during relaxing and recovering activities and decreases during stress. Accordingly, HRV is typically higher when the heart is beating slowly and decreases as the heart beats more quickly. In other words, heart rate and HRV have a generally inverse relationship. Also, HRV changes from day to day based on activity levels and amount of work-related stress. In addition to these external stress factors, internal stress factors cause variation daily HRV levels. Internal stress factors include poor nutrition, alcohol use, illness, etc.

The principle of the method is to utilize HRV and HR reactions as a tool for analyzing autonomic nervous system activity in order to build a digital model of human physiology for recognizing different bodily states.

The human nervous system consists of central nervous system and peripheral nervous system. The latter has two major divisions, the voluntary and the autonomic systems. The voluntary nervous system is concerned mainly with movement and sensation. The ANS mainly controls functions over which we have less conscious control. These include for example the cardiovascular system, whose regulation is fast and involuntary.

**Figure 3:**
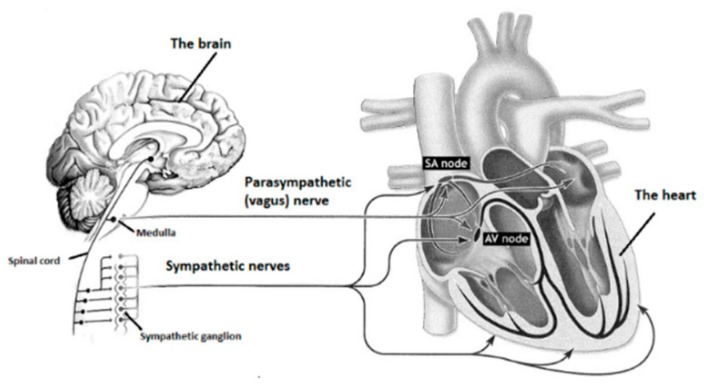
Autonomic nervous system controls different target organs via parasympathetic and sympathetic nerve cords. The parasympathetic nerve controlling the hearts is called the vagus nerve.

The ANS is divided into sympathetic and parasympathetic nervous systems (Figure 3). Sympathetic and parasympathetic nerve cords start from the central nervous system and lead to different target organs all around the human body. Sympathetic and parasympathetic divisions typically function simultaneously in opposition to each other. The parasympathetic division is primarily involved in relaxation, helping the body to rest and recover. The sympathetic division prepares the body to fight by accelerating bodily functions, and is also associated with stress.

With stress reactions, the human body tries to cope with the demands of the surrounding environment. Positive stress gives energy “to get the job done”. Negative stress causes negative emotions and reactions. Physiologically, the response to positive and negative stress is similar. As a result of the stress reaction, the ANS is activated and stress hormone production starts along with an increased rate and force of contraction of the heart [13]. The magnitude of the neuro-endocrine response reflects the metabolic and physiological demands required for the behavioral activity [14].

Therefore, although there is an ongoing debate of the exact definition of stress in the scientific literature, stress can be physiologically characterized by reduced recovery of the neuroendocrine reaction [14] and sympathetic dominance of the ANS function, whereas recovery is characterized as parasympathetic dominance.

In this study we will focus on HRV as an objective indicator to know the reactions to stress during the workday, as well as the quality of recovery of the nervous system (NS) during work, outside of work and at night. Its validity has been proven in several studies, both in the sports field with a focus on the study of high-performance training and in the workplace that concerns us [15,16,17,18,19]. In addition, a recent review of the literature on physiological biomarkers related to the study of stress in the workplace, concluded that the correlation between cortisol and work stress are less clear than those found with HRV [20].

In particular, the number of studies in which HRV has been used has been increasing in recent years. The most relevant findings show how acute stress correlates with a decrease in HRV during sleep [21] and during the day [22], finding a strong relationship between the decrease in HRV and work stress [5,12,18,23,24].

In order that the measurements made are accurate in a different environment to sports or medicine, much more accustomed to monitoring, it is necessary to take into account the technology to be implemented according to the characteristics of the population to be studied. Currently, there are several easy-to-use, mobile heart rate monitors that keep data of the intervals between beats (RR intervals) during the workday. Scientists to analyze HRV in sports science, medicine, and other fields of research have used commercial devices, such as heart rate monitors and activity clocks [25]. Recent studies have validated the HR recording devices in relation to different electrocardiogram systems with results that offer high reliability in the registration of RR interval series for the analysis of HRV similar to those offered by electrocardiography (ECG) equipment [26].

Among the new technologies applied in the workplace for HRV monitoring, we find the Body Guard 2 device of the Firstbeat^®^ philanthropy as one of the best options at the scientific level [15,16,17,18,19,20], since it also provides information about the quality of recovery of the records obtained.

In this sense, the quality of recovery is one of the key variables to take into account in stress management and not only the level of daily reaction to it. It should also be noted that the quality of recovery in the executive sector not only gives the duration and quality of sleep. The characteristics of the executive profile make it necessary to acquire personalized strategies that can help them to combat stress during the workday, outside the working day, and during the night, independently of the areas and activities that they have assigned and the variability of them. To this effect, studies indicate that HRV has been used to monitor the effort and recovery in sports such as judo or basketball [27,28], as well as after performing submaximal exercise [29]. However, there are few studies that analyze our target population specifically during the night to assess the quality of recovery; although there are studies carried out with Finnish workers (*n* = 16.275) that correlate a practice of high physical activity with lower percentages of stress during workdays and during one’s work, as well as a balance of stress. Also positively related to a lower BMI with better recovery during sleep [30], the correct design of healthy strategies within the workplace, sustainable over time, could help the quality of recovery of workers within and out of work.

For everything described above, the aim of this study has been to describe the physiological burden of stress and the quality of recovery in a population of senior managers during the workday analyzed during three different time zones: work, after work, and night.

As a secondary objective, we mark the relocation of the use of high-performance technology for the objectification of parameters related to health management and stress in executive positions of high responsibility in a business environment.

Our hypothesis is that the physiological load derived from a low-quality recovery during the 24 h working day negatively influences the increase in the physiological load that results from stress in the manager in the medium-long term.

## 2. Materials and Methods

### 2.1. Participants

The study was carried out with a total of 48 subjects (*n* = 48). Of this total, 28 were men and 20 were women. All of them are senior managers of multinational companies based in Spain with high categories of responsibility during the working day. The sample had an age of 45.92 ± 6.97 years, height of 1.72 ± 0.09 m, weight of 76.04 ± 13.57 kg, and BMI of 25.54 ± 3.39.

The sample analyzed was of Caucasian race with a medium-high socioeconomic status and payroll above the Spanish average (1636 €/month). In addition, during the period of analysis, the sample did not express situations of stress beyond normal to the researcher group, so that the records were made in situations of habitual stress to them, performing their daily tasks during the HRV registry.

All subjects were healthy subjects who had not suffered any type of cardiovascular or nervous system disease/disorder. The exclusion criteria for participation in the RR interval recordings included severe heart disease, very high blood pressure (≥180/100 mmHg), type 1 or 2 diabetes with autonomic neuropathy, severe neurological disease, fever or other acute illnesses, and a BMI > 40 kg/m^2^. These exclusion criteria represented by the manufacturer of the analysis software are presented in detail [31]. In all cases, acceptance and voluntary and informed signing of participation in the study was indispensable.

We certify that during the course of our investigation all the regulations marked on the ethical use of human volunteers marked in the Declaration of Helsinki were followed. In addition, the personnel in charge of collecting the data of the subjects included in the study did not include in the study data that could accurately identify the worker in accordance with the requirements of the General Data Protection Regulations (RGPD) (UE) 2016/679.

### 2.2. Material

**Figure 4:**
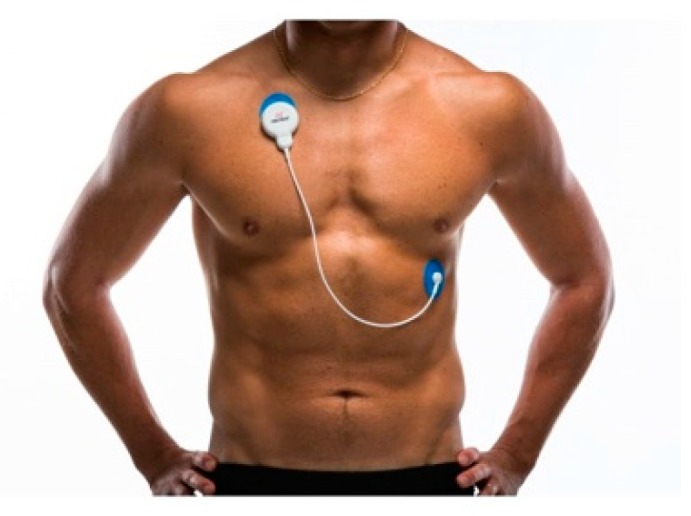
Firsbeat Bodyguard 2. Firstbeat Technologies Ltd.

The non-invasive device Firstbeat Bodyguard (Firstbeat Technologies Ltd., Jyväskylä, Finland) (Figure 4) was used to register the HRV. The data was recorded using Firstbeat Analysis Server software (version 6.3, Firstbeat Technologies Ltd.), which includes the artifact detection and correction function for irregular ectopic beats and signal noise. Its subsequent use was carried out with the exportation of the same to the software excel (version 15—year 2013) for the analysis of the individualized form of the relationship between stress and recovery (stress balance) in the different studied ranges: work, after work, and night, as well as the treatment of the different variables analyzed. All statistical analysis was performed with SPSS V.23. (SPSS Inc., Chicago, IL, USA) for Windows.

The device is used through two disposable electrodes. The hydrogel electrodes that were used during the data collection in our sample were the model Kendall 530 foam (Meditrace). All participants were given the device and six additional electrodes so that they could exchange them when showering, since the device cannot get wet.

### 2.3. Protocol

The measurement data was interpreted following the Healthy Box^®^ methodology for specialized research in companies (Freedom and Flow Company, Madrid, Spain). For the analysis of the data we took into account data such as sex, age, height, and weight for the interpretation of the results obtained through FirstBeat^®^. Ambulatory beat-to-beat R-R intervals were used to determine the amount and intensity of blood pressure (BP), stress, and recovery. The software calculates the HRV indices second by second using the short-time Fourier transform method and calculates the variables derived from HR and HRV of the respiration rate, oxygen consumption, on/off kinetics (increase or decrease of HR), and parameters describing the excess post-exercise oxygen consumption using neural networks. Subsequently, the software divides the measurement data into coherent data segments and categorizes these segments into different physiological states, such as physical activities (PA) of different intensities, stress, and recovery [32,33], taking into account the individual characteristics, levels, and scales of HR and HRV, and the individual relationships between HRV and autonomic control [34]. More information of this method of analysis is available in a document from Firstbeat Technologies Ltd [35].

All the participants received the device in hand in their respective offices, the protocol to be followed was explained to them during the next 48 h and they were given access to a diary where they indicated all the activities they would perform during the registration time, including working hours and sleeping time. All the participants had placed the device during 48 working hours with the objective of analyzing 24 full hours of registration, regardless of their lifestyle and working hours. This methodological decision was taken to try to homogenize to the maximum the different working hours that we can find in this studio population, where in some cases, the change of continental time between the different headquarters of the analyzed multinationals had to be taken into account.

### 2.4. Variables

To calculate the physiological load of stress and recovery we monitor through the Firstbeat^®^ Bodyguard 2 device we measure the HR in beats per minute (PPM), the VFC measured in seconds (seconds), and the analysis of Root Mean Square of the Successive Differences (RMSSD) as is measured in milliseconds (ms) during a workday. From these parameters, the variables analyzed were [33]:

RMSSD: one of the few time domain tools used to evaluate the variability of heart rate. RMSSD searches for the successive difference between the R-R intervals in order to calculate the square of the middle root of the junction of the adjacent R-R intervals and provide an indicator of the vagal cardiac control (parasympathetic tone). Therefore, we can say that it is a parameter that informs us of those variations that occur in a short period between the R-R intervals, obtaining information on how the parasympathetic nervous system (SNP) affects the cardiovascular system. Thanks to the RMSSD we know with little time of measurement if there have been changes, the greater the RMSSD being the greater the parasympathetic activity.

**Figure 5:**
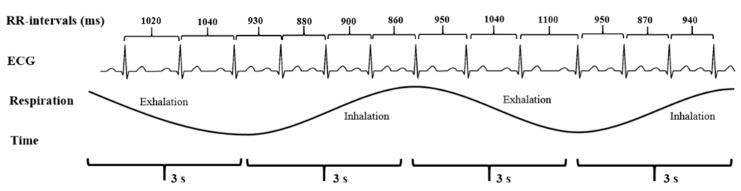
Electrocardiogram (ECG) exhibiting respiratory sinus arrhythmia.

Heart rate increases and, thus, the time between successive RR-intervals gets shorter during inhalation (inspiration) and longer during exhalation (expiration). This fluctuation in the time between the successive RR-intervals is called HRV. Image extracted by Stress and Recovery Analysis Methods based on 24 h heart rate variability, Firstbeat Technologies Ltd. (Figure 5).

- Time stress in 24 h (minutes): time in minutes of an average of 24 h reacting to stress.
- Stress percentage (%) (% Stress): percentage of reaction to stress in an average of 24 h.
- Recovery time in 24 h (minutes): total time, measured in minutes, in which the body is recovering.
- Percentage recovery (%) (% Recovery): percentage of total recovery time in 24 h.

**Figure 6:**
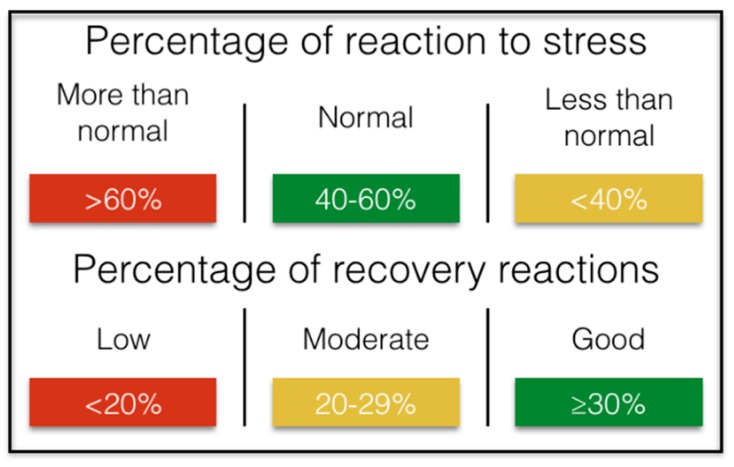
Values expressed in percentage of the impact of stress and recovery on performance.

The studies say that in 24 h, an adequate management of stress that impacts healthily in the professional and personal environment would entail between 40–60% of the time spent in activities that suppose a positive stress in the organism, indispensable for the high work performance, complemented with more than 30% of regenerative activities/recovery, which would be equivalent to 432 min of the 1440 min that day. We could summarize it (Figure 6):

It is important to note that stress is not always harmful. Stress can be positive or negative. The presence of stress may indicate that the person is experiencing something exciting or joyful. However, high stress indicators over a long period of time negatively impact personal well-being and health. Moderate stress levels during a normal working day are associated with high productivity at work.

Stress Balance: The 24 h stress balance features identify areas of stress in an executive’s routine. This variable evaluates the balance between stress and recovery during the day or time slots analyzed, identifying if the body tendency reacts predominantly towards stress or relaxation. 

Regular recovery is necessary for physiological systems to overcome the effects of stress. Recovery means reduced activation levels in the body in the absence of internal and external stress factors. During recovery, parasympathetic (vagal) activations dominate the ANS and psychophysiological resources are restored.

The connection between HRV and stress-recovery is commonly recognized. Strong indicators of recovery include individually low HR and high HRV. Night-time recovery rates are a key factor in stress balance.

The Firstbeat software gives us the stress balance value automatically for all the monitored record, this value being between 1 and −1 (values closer to 1 will indicate a correct recovery, while values close to −1 indicate a poor recovery) (Table 1).

Following the Healthy Box^®^ methodologyy for specialized research in companies (Freedom and Flow Company, Spain) and in order to homogenize the recorded data as much as possible, we decided to divide the day into three strips and analyze the variables in a segmented manner for each of them, protocolizing the analysis and data processing:

- Work: in those cases in which the subject did not indicate an atypical variation of his work schedule, we applied the protocol of the type schedule (09:00 a.m. to 6:00 p.m.), as well as in the subjects who specified us schedule, and we filtered all the minutes worked throughout the 24 h of analysis, even if it was a split day.
- After Work: we consider here all the hours in which you are not working or sleeping, being the standardized hours from 6:00 pm to 11:00 pm, always respecting the specifics of the sample indicated by each subject.
- Night: For the evaluation of the recovery index, the results are highly individualized, since the method is to use a 4 h window that starts 30 min after going to bed, in order to compare the intensity of recovery with the highest measurement of registry.

For the calculation of stress balance, all the moments in which there is no signal or identification by the device, were broken. In this way, we were able to calculate the stress balance in each time slot through the following formula:

$$\mathrm{Stress}\;\mathrm{Balance}\;=\;\frac{\left(\mathrm{Relaxion}+\mathrm{Recovery}\;\mathrm{from}\;\mathrm{exercise}\right)-“\mathrm{non}-\mathrm{identificable}”}{\left(\mathrm{Relaxion}\;+\;\mathrm{Recovery}\;\mathrm{from}\;\mathrm{exercise}\right)+“\mathrm{non}-\mathrm{identificable}”}$$

The last of the analyzed data was training effect (TE), which represents the degree of alteration of the homeostasis resulting from a physical activity session (in our case of the 24 h measurement). The effect of training is based mainly on the values of COPD during the exercise, which is extended depending on the physical state or level of activity of the people. The higher the value of TE, the greater the expected increase in maximum performance after the exercise will also be. COPD (and TE) increases when the intensity or duration of exercise increases.

- Training effect describes the effect of the exercise on a scale of 0–1: 0.0–0.9 = no effect; 1.0–1.9 = minor effect; 2.0–2.9 = maintenance effect; 3.0–3.9 = improvement effect; 4.0–4.9 = highly improving effect; 5.0 = temporary overreach effect.

### 2.5. Statistics Analysis

The data processing was carried out in two general lines:

- Descriptive study of the physiological burden of stress and recovery during a workday in a sample of *n* = 48 executives. All values are shown as mean ± standard deviation (Med ± DS).
- Descriptive study comparative of the physiological load of stress and recovery during a workday between men (*n* = 28) and women (*n* = 20).

All statistical analyses were performed with SPSS^®^ V.23. (SPSS Inc., Chicago, IL, USA) for Windows. All values are shown as mean ± standard deviation (Med ± DS). Due to the size of the sample, less than 50 subjects, the U-Mann Whitney test was carried out to verify the normality of the sample distribution. The level of significance was set at *p* < 0.05. Those variables that followed a normal distribution were applied the Student’s T test for independent samples with two-tailed analysis, making the comparison with a confidence interval of 0.95 percent. In case of finding differences, and in order to avoid the type I errors that are made when making multiple comparisons, the Bonferroni post hoc test was performed. For those variables that needed their corresponding analysis of non-parametric type, Friedman’s nonparametric test was used. To determine between which intervals differences were obtained, we used the nonparametric test of two Wilcoxon-related samples. The level of significance is set at *p* < 0.05. For the correlation tests, the Pearson or Spearman test was used according to parametric or nonparametric variables.

## 3. Results

In the descriptive study of the physiological burden of stress and recovery during a workday in a sample of *n* = 48 executives. All values are shown as mean ± standard deviation (Med ± DS).

The general aspects to consider are the reaction time to stress in minutes and percentage with respect to the total of the day. In this sense the reaction to stress can be positive or negative. An optimal average for work performance and health would be to find the percentage of reaction to stress between 40–60% of total hours of the day (9.6 h–14.4 h out of a total of 24 h). On the other hand, the relaxation time corresponds to the periods of time in which the body is recovering/regenerating. Important recovery periods include sleep and rest times during the day. As a reference, we should rest at least 30% (7.2 h) in a 24 h period. The result of the average percentage of time directly related to physiological reactions to stress during a 24 h workday in the group analyzed is 40.96 ± 18.91%, placing them within the values rated as habitual and optimal for performance. On the other hand, the average relaxation time in a 24 h period expressed as a percentage is 24.38 ± 11.45%, being lower than the recommended values (<30%). Significant differences have been found between stress time and relaxation time during the workday, a which mean that in a 24 h workday, stress is more present in the body than relaxation (*p* < 0.05). Below are the results analyzed for each time slot (Table 2).

As we can see in Table 2, the highest stress time is given in the time slot corresponding to the working hours, decreasing significantly in the after work range (*p* < 0.00) and during the night (*p* < 0.00). With respect to the relaxation time, we did not find significant differences in the time of relaxation inside and outside of work, although there we did between after work and night (*p* < 0.00), with other types of activities being able to exist outside the working hours that the device does not identify as stressors, and with it their corresponding impact on the SN, but that also do not allow the body to relax.

In the analysis of the impact of stress generated during working hours can generate in the rest of the variables, we found that there is a low positive correlation between the time of stress at work and stress time outside working hours (r = 0.30) (*p* < 0.05), being significantly different among them (*p* < 0.00), which indicates that the more stress at work, the more stress tends to be outside of working hours. We also found a low positive correlation between work stress and stress time present during the night time (r = 0.38) (*p* > 0.05), being significantly different, so, in this sense, having higher work stress is also relates to a greater tendency to suffer greater nighttime stress (*p* < 0.00). However, we did not find in the results any kind of correlation between the stress time shown during working hours and the nighttime relaxation time, which shows that the stress time generated during working hours does not have to involve lower values of relaxation during the night, although it does imply a significant decrease in the values of SB nocturne (r = −0.37) (*p* < 0.05).

On the other hand, when we analyze the impact of stress that is generated and/or remains outside of working hours after working hours, we find a moderate negative correlation between the stress time generated outside of working hours and the nighttime relaxation time, which indicates that the greater the hours of stress outside of work, the shorter the relaxation time during the night (r = −0.48) (*p* < 0.01) and the lower values of the SB at night (r = −0.35) (*p* < 0.05).

**Figure 7:**
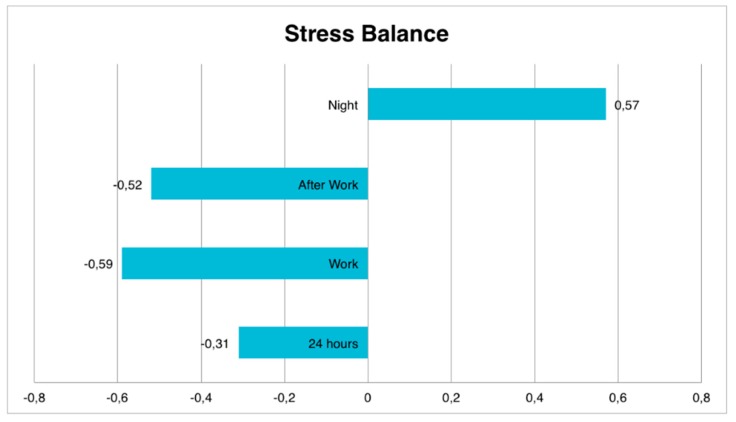
Stress balance for different time slots.

If we analyze separately the tendency of the organism to be in a state of stress or recovery during the full working day (24 h), the average found is identified with “moderate tendency to stress” (−0.31 ± 0.37), maintaining a high tendency to stress both within (−0.59 ± 0.48) and outside working hours (−0.52 ± 0.48), compensated with moderate recovery values only during the night (0.57 ± 0.43) (Figure 7).

In the descriptive study of the physiological load of stress and recovery during a workday, segmented by gender (men, *n* = 28 and women, *n* = 20), we only found significant differences between gender in the time of reaction to stress by the night, being significantly lower in men (62.37 ± 73.0 min) than in women (119.0 ± 98.5 min) (Table 3).

In the descriptive study of the effect of training during a working day, we found that the analyzed sample obtained a TE of 1.69 ± 0.67, which means that it has a low level of physical activity (less training effect).

## 4. Discussion

After analyzing the results, we explained that the main objective of this study was fulfilled, which was to describe the physiological burden of stress and the quality of recovery in a population of senior managers during the workday analyzed during three differentiated time slots: work, after work, and night. The marked secondary objective has also been met, through which it was sought to transfer the use of high-performance technology for the objectification of parameters related to health management and stress in executive positions of high responsibility to the business environment.

The hypothesis of research generated at the beginning of the study was fulfilled in which we established that the physiological load derived from a low quality of recovery during the 24 h workday, negatively influences the increase in the physiological load that stress entails in the manager in the medium-long term.

As we can see, the stress generated in the time interval between the work output and the time of going to bed is the one that significantly seems to influence more strongly in a low quality of recovery during the night since it does seem to influence the nighttime relaxation time with a negative correlation of r = −0.48. While the stress time generated during working hours does not correlate with it. In this case, whether in a general way or segmented by sex, the organism’s longest period of relaxation occurs significantly at night, finding in this range the only positive SB values, related to a better quality of recovery.

To this effect, the data show a low positive and significant correlation between stress time at work and stress time outside working hours, which a priori indicates that the more stress at work, the more stress tends to exist outside working hours, thus influencing a worse quality of night recovery. However, if the stress is significantly lower after working hours but does not find significant differences in the relaxation time inside and outside of work, it may lead us to think that there may be other types of activities outside of work hours that, although not being identified by the body as stressors and are not directly related to work, nor allow the body to relax.

In line with this hypothesis, the device used identifies physical activity as an element different from stress/relaxation, which is why we include in the analysis of variables the effect of training derived from the time that the device collects as a practice of physical activity. In the analyzed population, the effect of the training is 1.69 ± 0.67 out of a maximum of 5.0, which means that they hardly did any physical activity during the day they wore the device, so we can rule out the practice of physical activity outside the working hours as a physical stressor responsible for the values recorded with respect to the time of stress and its relationship with relaxation time outside working hours, leaving the hypothesis open to the cause of stress outside working hours corresponds to other types of activities personal and/or professional.

In this regard, the study by Föhr et al., (2016) correlated in a sample of 16,275 lower Finnish workers percentages of stress during the days of work and during the work itself in those who had a practice of high daily physical activity, impacting also in its quality of recovery [28], In the upper management segment, the practice of physical activity is, thus, defined as a great compensatory tool for the stress generated during the day, deficient in the sample analyzed, which would serve to improve not only their levels of strength and aerobic capacity, but also improve the effects of training in general health and the management of physiological stress in particular.

On the other hand, our results confirm previous studies showing a strong relationship between the decrease in HRV and stress in the body [20,23,24], however most of these studies describe stress as “work-related stress”. Analyzed in a general way, nevertheless, by segmenting the working day in three time zones—work, after work, and night—our results show how the stress generated during working hours is not directly responsible for the decrease in HRV sustained in time, and may not be in this case and for this sample, the problem to “manage” if the period included outside work hours (after work) until the time of going to bed.

Acute stress correlates with a decrease in HRV during sleep [21] and during the day [22], which may mean that the acute stress peaks that significantly decrease the quality of recovery during the night in our group are not given during work hours, but outside of work.

In this sense, the management of stress produced outside of work is decisive to achieve a quality of positive recovery and enough to compensate the reactions to stress the rest of the workday, if the stress generated outside of work is not caused for the practice of physical activity (positive stress), there are other personal or professional causes that strongly condition the quality of recovery in the upper management segment. In relation to this line of hypotheses, the team of Zoupanou et al. (2013) concluded in its study that employees strongly influenced by work are the ones with the highest negative stress, however, this result cannot be confirmed with our study, being necessary to continue expanding the sample with the aim of knowing which activities are those that cause an increase or maintenance of stress levels outside working hours, and cannot be directly attributed solely to the workload generated during the same.

## 5. Conclusions

The stress generated or maintained outside working hours correlates significantly with a lower quality of recovery during the 24 h workday.

It is necessary to prioritize strategies that help improve stress management in executives through the improvement of tools and strategies that promote greater relaxation outside working hours mainly.

The sample analyzes presents values of physical activity practice lower than those recommended by the World Health Organization.

## 6. Limitations

Variables, such as the IQ or socioeconomic status throughout the working life of the sample, were not analyzed in this study, with possible covariates that would improve the analysis.

The comparison of the stress obtained through HRV with self-perceived stress questionnaires could be very useful to obtain the physiological and mental relationship of stress both at a specific time of the day and a total balance throughout the same.

There is some limitation in the exact control of the work and after work bands in terms of the completion of the work moment and the start of after work, which can be a factor of confusion for the reader. The rest of the strips selected in the sample are very clear and precise. When developing the sample we analyzed its functions in a fixed physical place, and we could assess the possibility that these strips were inside the workplace and outside the workplace, as long as there was no mobility on the part of the subject.

## Figures and Tables

**Table 1:** Stress balance areas.

High Tendency to Stress	Moderate Tendency to Stress	Moderate Tendency to Relaxation	High Tendency to Relaxation
From −1 to −0.5	From −0.49 to 0	From 0.1 to 0.49	From 0.5 to 1

**Table 2:** Descriptive analysis of the physiological load and recovery (Med ± SD).

	Relax Time (min)	%Relax Total	Stress Time (min)	%Stress Total	Stress Balance
Work	51.38 ± 89.14	3.57 ± 6.19%	310.11 ± 157.84 *	21.54 ± 10.96%	−0.59 ± 0.48 *
After Work	38.41 ± 45.67	2.67 ± 3.17%	212.89 ± 93.08 *	14.78 ± 6.46%	−0.52 ± 0.48 *
Night	276.13 ± 94.88 *	19.18 ± 6.59% *	85.78 ± 88.11 *	5.96 ± 6.12%	0.57 ± 0.43 *
Total	351.75 ± 164.86	24.38 ± 11.45%	589.88 ± 272.37	40.96 ± 18.91%	−0.31 ± 0.37

Min = minutes. * *p* < 0.01

**Table 3:** Descriptive analysis of the physiological load and recovery segmented by sex (Med ± SD).

		Relax Time (min)	% Relax Total	Stress Time (min)	% Stress Total	Stress Balance
Work	Men	60.78 ± 104.02	4.22%	313.56 ± 175.72	21.78%	−0.63 ± 0.48
	Women	38.70 ± 64.33	2.69%	305.45 ± 134.25	21.21%	−0.55 ± 0.50
After work	Men	42.37 ± 45.84	2.94%	194.48 ± 90.32	13.50%	−0.46 ± 0.53
	Women	32.79 ± 46.07	2.28%	239.05 ± 92.98	16.60%	−0.61 ± 0.38
Night	Men	292.3 ± 90.79	20.30%	62.37 ± 73.05 *	4.33%	0.65 ± 0.43
	Women	253.16 ± 98.23	17.58%	119.05 ± 98.53 *	8.27%	0.47 ± 0.41
Total	Men	381.32 ± 182.87	26.48%	550.04 ± 294.57	38.20%	−0.23 ± 0.41
	Women	310.35 ± 128.91	21.55%	645.65 ± 233.66	44.84%	−0.41 ± 0.26

* *p* > 0.05.
